# Prediction of Early- and Late-Onset Pre-Eclampsia in the Preclinical Stage via Placenta-Specific Extracellular miRNA Profiling

**DOI:** 10.3390/ijms24098006

**Published:** 2023-04-28

**Authors:** Angelika V. Timofeeva, Ivan S. Fedorov, Yuliya V. Sukhova, Tatyana Y. Ivanets, Gennady T. Sukhikh

**Affiliations:** 1 Kulakov National Medical Research Center of Obstetrics, Gynecology, and Perinatology, Ministry of Health of Russia, Ac. Oparina 4, 117997 Moscow, Russia; 2 Department of Obstetrics, Gynecology, Perinatology and Reproductology, First Moscow State Medical University Named after I.M. Sechenov, 119991 Moscow, Russia

**Keywords:** early pre-eclampsia, late pre-eclampsia, miRNA, small RNA deep sequencing, quantitative PCR in real-time, placenta, blood serum, first trimester

## Abstract

Pre-eclampsia (PE) is one of the severe complications of pregnancy in 3–8% of all cases and is one of the leading causes of maternal and perinatal mortality. The fundamental role in the pathogenesis of PE is assigned to maternal and/or placental factors, whereby the combination and manifestation of which determines the time of onset of the clinical symptoms of PE (before or after 34 weeks of gestation) and their severity. It is known that the expression level of miRNAs, the regulators of signaling cascades in the cell, depends on gestational age. In the present study, we focused on the identification of the placenta-specific miRNAs that differentiate between early- and late-onset pre-eclampsia (ePE and lPE) throughout pregnancy, from the first to the third trimester. A total of 67 patients were analyzed using small RNA deep sequencing and real-time quantitative PCR, which resulted in a core list of miRNAs (let-7b-5p, let-7d-3p, let-7f-5p, let-7i-5p, miR-22-5p, miR-451a, miR-1246, miR-30e-5p, miR-20a-5p, miR-1307-3p, and miR-320e), which in certain combinations can predict ePE or lPE with 100% sensitivity and 84–100% specificity in the 1st trimester of pregnancy. According to the literature data, these miRNA predictors of PE control trophoblast proliferation, invasion, migration, syncytialization, the endoplasmic reticulum unfolded protein response, immune tolerance, angiogenesis, and vascular integrity. The simultaneous detection of let-7d-3p, miR-451a, and miR-1307-3p, resistant to the repeated freezing/thawing of blood serum samples, in combination with biochemical (b-hCG and PAPP-A) and ultrasound (UAPI) parameters, allowed us to develop a universal model for the prediction of ePE and lPE onset (FPR = 15.7% and FNR = 9.5%), which was validated using a test cohort of 48 patients and demonstrated false-positive results in 26.7% of cases and false negatives in 5.6% of cases. For comparison, the use of the generally accepted Astraia program in the analysis of the test cohort of patients led to worse results: FPR = 62.1% and FNR = 33.3%.

## 1. Introduction

Pre-eclampsia (PE) is a multisystem complication in 3–8% of all pregnancies [1] that accounts for 16–18% of maternal and 40% of fetal and neonatal deaths [2]. According to the International Society for the Study of Hypertension in Pregnancy (ISSHP), PE is defined as de novo hypertension (blood pressure above 140/90 mm Hg) after 20 weeks of gestation, accompanied by proteinuria (at least 0.3 g/L per day) or signs of acute renal failure, liver dysfunction, neurological disorders, hemolysis, thrombocytopenia, or intrauterine growth retardation. PE may be early (ePE) or late (lPE), depending on the time of onset of clinical symptoms (before or after the 34th week of pregnancy, respectively) [3]. In addition, ePE is characterized by a more severe course, and accounts for 5–20% of all types of PE. Adverse outcomes for the fetus are associated with chronic hypoxia and a high frequency of developmental delay, and also cause complications in the fetus due to prematurity, including respiratory distress syndrome, infectious and inflammatory diseases, intraventricular hemorrhages, cerebral palsy, cognitive retardation, autism, psychomotor and behavioral disorders, and/or learning disabilities [4,5].

Maternal and/or placental factors play a fundamental role in the pathogenesis of PE, which determines the time of onset of clinical manifestations and their severity. The impairment of trophoblast cell proliferation and differentiation in the pre-implantation stage in case the embryonic program runs with errors, and in subsequent stages of implantation due to inflammatory changes in the decidual layer, may affect the interaction of trophoblast and endometrial cells and subsequent placentogenesis [6,7,8]. Impaired cell differentiation of the extravillous trophoblast leads to the insufficient remodeling of the spiral uterine arteries: first, this occurs in the decidual segment before the 10th week of pregnancy in the form of reduced arterial obstruction via endovascular trophoblast cells and, as a result, there is damage to the placental villi via reactive oxygen and nitrogen species [9], and then it occurs in the segments of the myometrium from the 16th to the 18th week of pregnancy [10]. The result of the abnormal restructuring of the uterine arteries is an increase in their resistance and mechanical damage of the placental villi due to increased blood pressure entering the intervillous space [11,12,13,14]. The failure of the utero-placental blood flow occurs, which leads to hypoxic/ischemic changes in the placental tissue [1,15]. Various biological factors are released from the ischemic placenta, causing the systemic damage of the vascular endothelium and the occurrence of acute multiple organ failure in the mother. Decreased concentrations of circulating placental factors such as pregnancy-associated plasma protein A (PAPP-A) and placental growth factor (PlGF), as well as an increase in the formation of soluble fms-like tyrosine kinase-1, levels of vascular endothelial growth factor A (VEGF-A), inhibin A, activin A, procoagulant P-selectin, pro-inflammatory interleukin 2, and tumor necrosis factor alpha, are associated with PE [1,16,17,18]. Maternal pathogenetic factors include genetic predisposition, immunological factors, metabolic syndrome, and diabetes mellitus; chronic arterial hypertension can aggravate the maternal susceptibility to factors secreted by ischemic placental tissue and accelerate the onset of clinical symptoms in the mother [19].

Taking into account the role of epigenetic mechanisms regulating the differentiation, migration, and invasion of trophoblast cells [20], whereby one of the main components of which is represented by small non-coding RNAs (sncRNAs), numerous studies have been carried out to assess the qualitative and quantitative composition of miRNAs in women with PE compared to physiological pregnancy. MiRNAs are small (about 20–24 nucleotides long) single-stranded molecules that down-regulate gene expression at the post-transcriptional level by destabilizing mRNA and/or inhibiting protein translation. During pregnancy, miRNAs can control the invasion and migration of trophoblast cells and angiogenesis, in particular by regulating the expression levels of VEGF, sFlt-1, and HIF-1α [21,22]. Specific miRNA expression patterns were found in the placenta and peripheral blood of pregnant women with PE [23,24]. Winger E. et al. analyzed the predictive potential of the expression levels of 30 miRNAs in the peripheral blood leukocytes of women to predict the development of PE in the 1st trimester of pregnancy via quantitative PCR [25]. In addition, the composition of miRNAs in the blood plasma of pregnant women was analyzed, and it was found that a third of 368 identified miRNAs were not packed into exosomes; only 8 exosome miRNAs (miR-134, miR-196b, miR-302c, miR-346, miR-376c, miR-486-3p, miR-590-5p, and miR-618) significantly differed in PE from physiological pregnancy at the time of delivery, and of these, only 4 miRNAs (miR-134, miR-376c, miR-486-3p, and miR-590-5p) predicted the development of PE in the 1st trimester of pregnancy [26]. Gromadnikova I. and colleagues assessed the potential of miR-516b-5p, miR-517-5p, miR-518b, miR-520a-5p, miR-520h, and miR-525-5p in the blood plasma exosomes of pregnant women for the prediction of PE and gestational hypertension during the first-trimester screening [27]. Caterina Licini revealed an early circulating biomarker of PE-miR-125b, which targeted the surface antigen of trophoblast cells ((Trop)-2) and was involved in the regulation of intercellular adhesion and cell proliferation [28].

Despite the revealed predictive significance of certain miRNAs in diagnosing the development of PE in the preclinical stage, the recent investigations have only been devoted to lPE, but not to ePE, which is characterized by a more severe course with unfavorable maternal and perinatal outcomes. In connection with the above, the purpose of this study was to identify placental tissue-specific miRNA molecules that have prognostic significance in assessing the likelihood of developing ePE and lPE by analyzing the blood serum of women at 11–14 weeks of gestation using the deep sequencing method, with subsequent validation via real-time quantitative PCR.

## 2. Results

### 2.1. Search for Placenta-Specific Extracellular miRNAs in ePE and lPE at the Time of Delivery

In the first stage of the study, the first cohort of patients (Table 1) was analyzed to identify the miRNAs that differentiate between ePE and lPE according to the expression profile in the placenta and peripheral blood plasma of patients at the time of delivery. The comparison group for the analysis of the ePE included patients who delivered before 34 GW (N < 34) due to a lack of a possibility of prolonging the pregnancy. The comparison group for the analysis of the lPE included patients with a physiological pregnancy (N > 34). Taking into account the possible influence of various complications of pregnancy, leading to its termination, on the miRNA profile, we conducted a careful selection of patients to form a control group: “N < 34”. The group “N < 34” for the analysis of the placenta and peripheral blood plasma of pregnant women with ePE included patients with normal screening data of the 1st and 2nd trimester of pregnancy, and normal parameters of feto-placental and utero-placental blood flow, but urgent delivery at 25–32 weeks due to the thinning of the scar on the uterus up to 1–1.2 mm according to ultrasound (a thorough defect in the scar area was not determined) after the previous 1–3 caesarean sections, in combination with or without isthmic-cervical insufficiency and the onset of regular labor; with data on clinical and laboratory research methods that were within the normal range; with all fetal membranes intact at delivery, and whereby the outflowing amniotic fluid was light; and where no signs of chorioamnionitis were observed. No pathological changes in the morphological structure of the placenta were found in any patient from group “N < 34”. However, we found statistically significant differences in 305 out of 577 identified miRNAs in the placental tissue when comparing the miRNA sequencing data in the control groups (N > 34 vs. N < 34, Table S1, Sheet 1) which precisely indicates gestational changes in miRNAs expression, emphasizing the importance of the comparison of the ePE or lPE group with the control group of the corresponding gestational age (N < 34 and N > 34, respectively). At the same time, when comparing the miRNA sequencing data in the blood plasma of women from the control groups (N > 34 vs. N < 34), no statistically significant change in the content of any miRNA was found (Table S1, Sheet 1).

**Figure 1:**
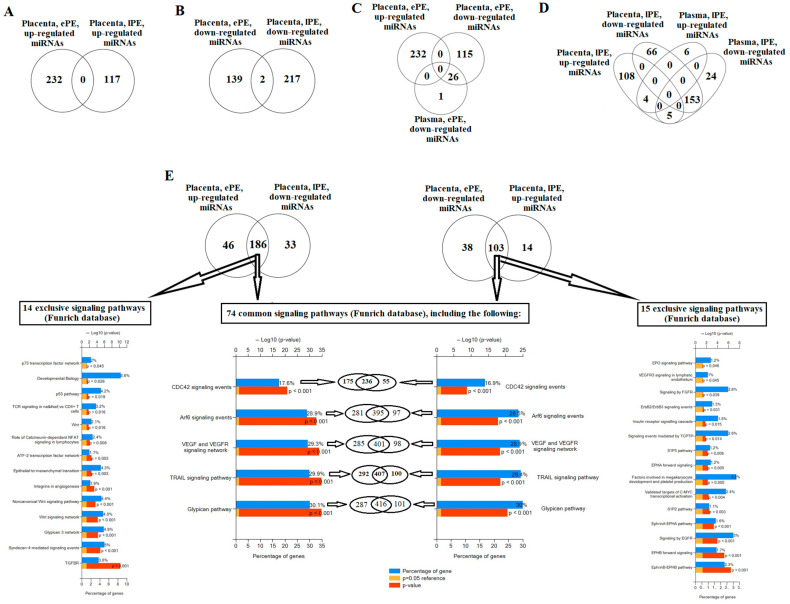
Comparison of differentially expressed miRNAs in the placenta and blood plasma of pregnant women with ePE and lPE at the time of delivery via the plotting of Venn–Euler diagrams and the analysis of the signaling pathways regulated by them in the Funrich program. (**A**) Search for intersection of lists of significantly up-regulated miRNAs in the placenta of those with ePE and lPE. (**B**) Search for intersection of lists of significantly down-regulated miRNAs in the placenta of those with ePE and lPE. (**C**) Search for intersections of lists of differentially expressed miRNAs in the placenta and blood plasma of women with ePE. (**D**) Search for intersections of lists of differentially expressed miRNAs in the placenta and blood plasma of women with lPE. (**E**) Signaling pathways regulated by the miRNAs with multidirectional changes in the expression levels of placental tissue in those with ePE and lPE.

miRNA profiling in the placental tissue and peripheral blood plasma of the patients of the analyzed groups was carried out using NGS. Fold changes in the miRNA expression level are presented in Table S1 (Sheet 2—for ePE placenta; Sheet 3—for lPE placenta; Sheet 4—for ePE blood plasma; and Sheet 5—for lPE blood plasma). While comparing the lists of differentially expressed miRNAs in the placental tissue of those with ePE and lPE, their intersection was either absent in the case of up-regulated miRNAs (Figure 1A), or was minimal in the case of down-regulated miRNAs (Figure 1B). It is important to note that the vast majority of miRNAs associated with ePE or lPE present in maternal circulation are placenta-specific and have a reduced level (Figure 1C and 1D, respectively). Moreover, in the case of ePE, no elevated miRNAs were detected in blood plasma (Figure 1C), whereas in the case of lPE, 10 miRNAs had elevated levels, and of which 4 miRNAs were of placental origin (Figure 1D). An important finding of the present study was the existence of multidirectional changes in the expression level of most miRNAs in ePE and lPE in placenta tissue (Figure 1E): 186 miRNAs (List 1) were up-regulated in ePE and down-regulated in lPE, whereas 103 miRNAs (List 2) were down-regulated in ePE and up-regulated in lPE. The obtained results reflect the pathogenetic differences between ePE and lPE at the molecular level. Therefore, it seemed interesting to us to analyze the signaling pathways influenced by these miRNAs with multidirectional changes in expression levels, as presented in Table S2 (186 miRNAs, List 1) and in Table S3 (103 miRNAs, List 2). Using the Funrich “miRNA enrichment” algorithm, 74 target signaling pathways were found to be common for the two lists of miRNAs (Figure 1E), but the protein products of the target genes of these miRNAs, forming each of the 74 signaling pathways, were either the same or different for List 1 and List 2 miRNAs, as demonstrated for the “CDC42 signaling events”, Arf6 signaling events, VEGF and VEGFR signaling networks, TRAIL signaling pathway, and Glypican pathway. That is, the miRNAs of different types from Lists 1 and 2, due to the different direction of changes in the expression level in the case of early PE or late PE, have opposite effects on the target genes of the same signaling pathway, and the activity of which depends on the preferred effect of each miRNA. In addition, 14 signaling pathways were identified that were only regulated by miRNAs from List 1, but not List 2 (in particular, the epithelial-to-mesenchymal transition, Wnt signaling pathway, and non-canonical signaling pathway), along with 15 signaling pathways regulated by miRNAs only from List 2, but not List 1 (in particular, the EphrinB-EPHB pathway, the EphrinA-EPHA pathway, the factors involved in megakaryocyte development and platelet production, and the EPO signaling pathway). Obviously, the differences in the pathogenesis of ePE and lPE were amplified by the presence of differentially expressed miRNAs specific to 1 of the 2 forms of PE: 46 up-regulated and 33 down-regulated miRNAs for ePE, and 38 down-regulated and 14 up-regulated miRNAs for lPE (Figure 1E).

Given the magnitude of the changes in the functioning of the signaling pathways with an imbalanced expression of the miRNAs influencing them in early and late PE, it is obvious that it is impossible to look for differences in the pathogenesis of the two forms of pre-eclampsia by analyzing any one signaling pathway. A hint toward the possibility of cascade changes in the activity of signaling pathways in the placenta is a change in the content of placenta-specific miRNAs in the blood plasma of pregnant women. Moreover, it is important to search for miRNAs associated with ePE and lPE, differentially expressed both in the preclinical stage of the manifestation of the disease and at the time of delivery in comparison with uncomplicated pregnancy. Therefore, the next stage of our study compared the obtained miRNA expression profiles in the placenta and blood plasma at the time of delivery with those in the blood serum of women in the first trimester of pregnancy.

### 2.2. Retrospective Analysis of miRNA Expression Profile in the Blood Serum from Women in the First Trimester of Pregnancy

A retrospective analysis of the miRNA expression profiles in the blood serum of the second cohort of 40 patients aged from 27 to 40 years in a period of 11–14 GW after receiving information about the clinical diagnosis at the time of delivery (Table 2) was carried out via deep sequencing to identify the miRNAs associated with the development of ePE and lPE in the preclinical stage. Four groups of patients were formed: (1) ten women with low risk of PE (according to the Astraia program) and physiological course of full-term pregnancy (N); (2) nine women with high sFLT-1/PLGF ratio in the second and third trimesters of full-term pregnancy without signs of PE (Nhr); (3) ten women with manifestation of PE at 34–37 GW (lPE); and (4) eleven women with manifestation of PE at twenty-five–thirty-three GW (ePE).

**Figure 2:**
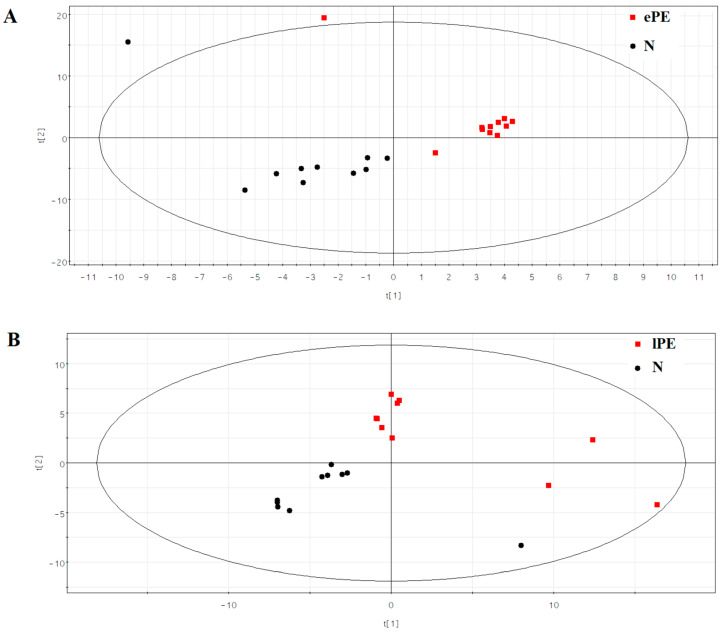
PLS analysis of NGS data on read counts of miRNAs in the blood serum samples from patients of the second cohort. (**A**) The arrangement of blood serum samples from women with ePE and without PE (N). (**B**) The arrangement of blood serum samples from women with lPE and without PE (N).

Using the Deseq program, a statistically significant increase in the expression levels of hsa-miR-16-5p (*p* = 0.00048) and hsa-miR-125b-5p (*p* = 0.01674) was revealed in the ePE group relative to group N (Table S4, Sheet 1), and the lPE group relative to the N group showed an increase in the levels of hsa-miR-221-3p (*p* = 0.00009), hsa-miR-146a-5p (*p* = 0.00024), hsa-miR-222-3p (*p* = 0.00075), hsa-miR-21-5p (*p* = 0.00162), hsa-miR-199a-3p (*p* = 0.00464), hsa-miR-199b-3p (*p* = 0.00481), hsa-miR-199a-5p (*p* = 0.00585), hsa-miR-30c-5p (*p* = 0.02807), and hsa-miR-29a-3p (*p* = 0.03146), and a decrease in the level of hsa-miR-1292-5p (*p* = 0.01780) (Table S4, Sheet 2). Because of the small number of miRNAs that significantly distinguished PE from N and the lack of overlap between the lists of differentially expressed miRNAs in ePE and lPE, we considered it inappropriate to develop logistic regression models based on the content of these molecules in blood serum in order to assess their prognostic significance in the genesis of PE. Moreover, it is desirable that these miRNAs are placenta-specific, implying that an imbalance in expression reflects abnormalities in the activity of signaling pathways in the placenta leading to the development of ePE or lPE. If one finds a common list of miRNAs involved in the pathogenesis of both ePE and lPE, but having different contributions to the further occurrence of clinical manifestations of PE, it will be technically easier to perform the diagnostic test. For this purpose, the contribution of each identified miRNA to the separation of samples according to the presence or absence of ePE (Figure 2A) and lPE (Figure 2B) was evaluated using partial least squares (PLS) regression.

In Figure 2A,B, one can see the arrangement of the samples with the formation of clearly spatially separated clusters as black symbols (normal) and red symbols (pre-eclampsia), where miRNAs with a variable importance in projection (VIP) score > 1 have the greatest contribution to this separation (Table S5). It should be noted that some of the miRNAs listed in Table S5 showed statistically significant correlations in their content in the blood serum of pregnant women with the average levels of blood pressure, β-hCG, PAPP, and uterine artery pulsation index according to the screening data of the first trimester of pregnancy (Table 3 and Table 4).

The search for interactions between miRNAs and genes associated with a disease (data obtained from Alliance of Genome Resources) was carried out using the miRWalk database (http://mirwalk.umm.uni-heidelberg.de/diseases/ (accessed on 28 January 2023)). We found that most of the miRNAs correlated with the mean blood pressure levels in pregnant women influence the expression of target genes involved in the occurrence of essential hypertension, renovascular hypertension, and pre-eclampsia (Table 3).

**Figure 3:**
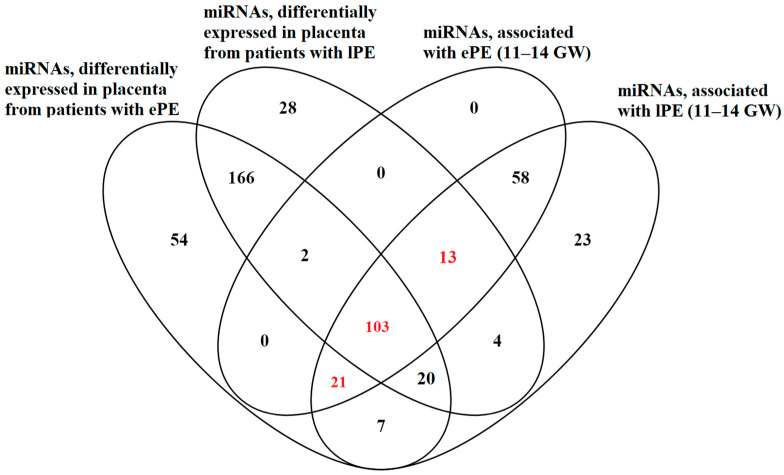
Venn–Euler diagram of circulating miRNAs associated with PE at 11–14 GW and differentially expressed miRNAs in placenta from patients with PE at the time of delivery.

Table 4 shows that miRNAs correlating with the UAPI, b-hCG, and PAPP-A can modify the expression of the target genes involved in the occurrence of vascular disease, placental insufficiency, and pre-eclampsia, according to the miRWalk database. To further consider only placenta-specific miRNAs among the molecules listed in Table S5, a Venn–Euler diagram was plotted (Figure 3), and the intersection of the following lists of miRNAs was identified: “differentially expressed miRNAs in placenta from patients with ePE at the time of delivery” (Table S1, Sheet 2), “circulating miRNAs associated with ePE at 11–14 GW” (Table S5), “differentially expressed miRNAs in placenta from patients with lPE at the time of delivery” (Table S1, Sheet 3), and “circulating miRNAs associated with lPE at 11–14 GW” (Table S5).

Figure 3 shows that all miRNAs circulating in the blood and associated with ePE at 11–14 GW are specific to the placenta. Among the circulating miRNAs associated with lPE at 11–14 GW, most are placenta-specific, but the remaining 23 out of 249 miRNAs are of non-placental origin. A total of 137 placenta-specific miRNAs identified in the blood serum in the first trimester of pregnancy and associated with ePE and lPE (highlighted in red in Figure 3) were considered for further analysis. Using a script written in the R-system to develop logistic regression models, various combinations of 137 miRNAs were analyzed, whereby the content of which in blood serum at 11–14 GW identifies patients with a high risk of developing ePE and lPE according to NGS data. The different combinations of 4 miRNAs with the highest predictive value (AUC = 1) are presented in Table S6 (Sheet 1).

**Figure 4:**
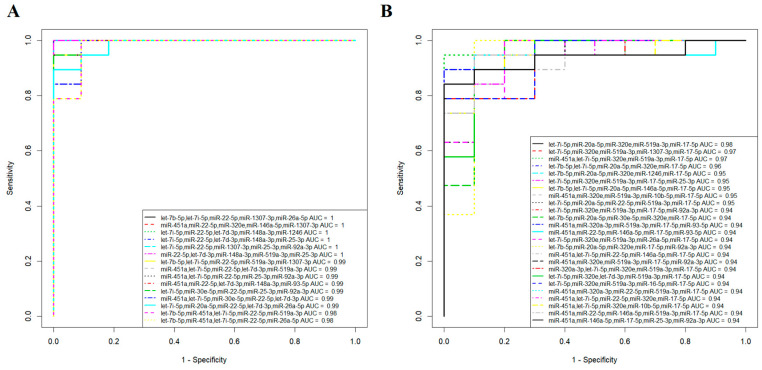
Logistic regression models for predicting ePE (**A**) and lPE (**B**) in the first trimester of pregnancy according to the miRNA profiles in the blood serum of patients. Quantitative RT-PCR data.

NGS data were validated via quantitative real-time RT-PCR using cel-39 (Qiagen) as the reference RNA. We selected 17 miRNAs (let-7b-5p, miR-451a, miR-320a-3p, let-7f-5p, let-7i-5p, miR-20a-5p, miR-30e-5p, miR-22-5p, miR-320e, let-7d-3p, miR-146b-5p, miR-148a-3p, miR-519a-3p, miR-99a-5p, miR-1307-3p, miR-26a-5p, and miR-1246), which in different combinations predict the development of both ePE and lPE. Based on the obtained qPCR data (-∆Ct values) presented in Table S6 (Sheet 2), logistic regression models were developed to predict ePE (Figure 4A) and lPE (Figure 4B), while comparing them with samples from the N and Nhr groups. Combining the two normal groups (N and Nhr) to form a comparison group was necessary to consider the cases where the risk of PE in terms of the sFLT/PLGF ratio was high, but the clinical signs of PE did not start until delivery.

The first three combinations of miRNAs used to develop the logistic regression models are shown in the inset of Figure 4A and were able to predict the development of ePE in the 1st trimester of pregnancy (*p* < 0.00001, threshold = 0.5) with 100% sensitivity and 100% specificity. The best predictive value in assessing the likelihood of developing lPE in the 1st trimester of pregnancy was provided via the combined detection of let-7i-5p, miR-320e, miR-519a-3p, miR-1307-3p, and miR-17-5p (84% specificity, 100% sensitivity, *p* < 0.0001, threshold = 0.159) or a combination of miR-451a, let-7i-5p, miR-320e, miR-519a-3p, and miR-17-5p (95% specificity, 100% sensitivity, *p* < 0.00001, threshold = 0.4628) in the logistic regression models of Figure 4B, i.e., lPE was overdiagnosed in 16% or 5% of cases, respectively, while no lPE cases were missed (FNR = 0).

**Figure 5:**
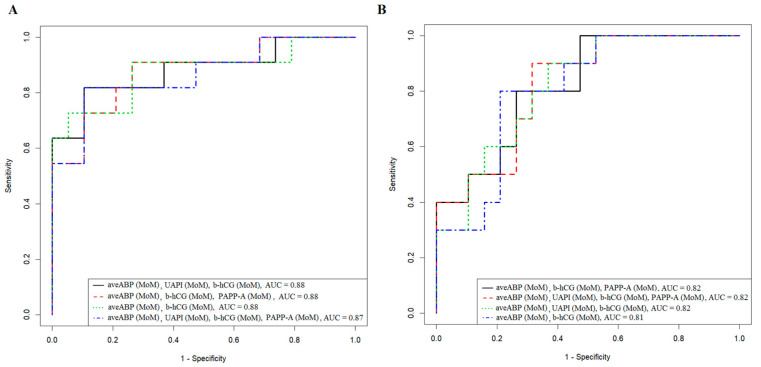
Logistic regression models for predicting early PE (**A**) and late PE (**B**) in the first trimester of pregnancy according to biochemical and ultrasound data.

To assess the prognostic significance of biochemical parameters (b-hCG, b-hCG (MoM), PAPP-A, PAPP-A (MoM)), fetal ultrasound data (crown-rump length, CRL), nuchal translucency thickness (NT), uterine artery pulsatility index (UAPI), UAPI (MoM), and average arterial blood pressure (aveABP, MoM) during the first-trimester screening, logistic regression models were developed to predict the likelihood of ePE (Figure 5A) and lPE (Figure 5B) onset while analyzing the second cohort of patients.

The best model for predicting ePE and lPE in the 1st trimester of pregnancy with the lowest percentage of missed PE cases (FNR = 5.3% and FNR = 21%, respectively) was the combined assessment of aveABP (MoM) and b-hCG (MoM), leading to false-positive results in 27.3% and 20% of cases in the diagnosis of ePE and lPE, respectively. The combined determination of aveABP (MoM), UAPI (MoM), b-hCG (MoM), and PAPP-A (MoM) reduced the overdiagnosis of ePE (FPR = 18.2%) and lPE (FPR = 10.0%) but increased the number of cases of missed ET (FNR = 10.5% and FNR = 31.6%, respectively).

**Figure 6:**
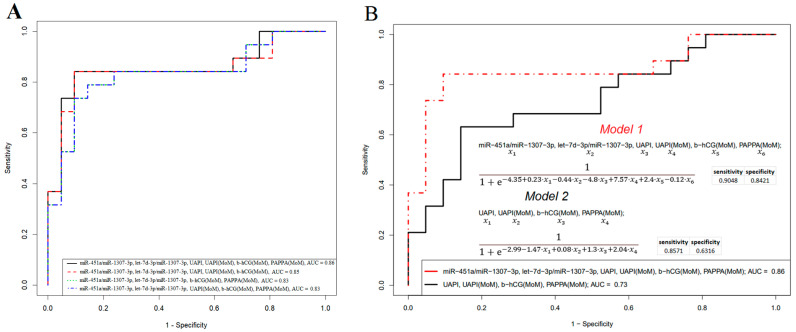
Logistic regression models for predicting PE in the first trimester of pregnancy according to biochemical and ultrasound parameters and miRNA levels. (**A**) Models based on the combination of biochemical and ultrasound parameters and miRNA levels. (**B**) Comparison of two models based on the biochemical/ultrasound parameters with or without the miRNA level dataset.

In the case of using a quantitative analysis of miRNA in blood serum (Figure 4A,B) as a potential additional method for predicting the development of PE in women undergoing biochemical and ultrasound examinations as part of first-trimester screening, it is necessary to reduce the number of miRNAs that form a combination in developed logistic regression models to reduce the cost of a comprehensive study. Moreover, the normalization of quantitative RT-PCR data for each miRNA to the external control cel-39 (Qiagen) does not consider the possible degradation of RNA before the stage of its isolation from the biological sample, since it is introduced into the sample in the stage of phenol extraction. For miRNA quantification, an endogenous control is preferable. In addition, to simplify the procedure for testing samples, a logistic regression model is needed that is the same for predicting both ePE and lPE. Therefore, it was decided, as an alternative option, to build a logistic regression model involving biochemical and ultrasonic parameters and to add two or three miRNA molecules to this model to improve its characteristics. miR-451a, let-7d-3p, and miR-1307-3p were chosen for this purpose for two main reasons: their participation in logistic regression models for ePE and lPE prediction and the absence of a decrease in their concentration in the blood serum sample during double freeze/thaw cycles, unlike other miRNAs. The concentration of the miR-451a, let-7d-3p, and miR-1307-3p in the blood serum samples did not decrease during the double freeze/thaw cycles, probably because of their ability to pack into exosomes, that we have demonstrated via the deep sequencing of small non-coding RNAs from exosomes obtained via culturing human mesenchymal stromal cells (54825 read counts for miR-451a and 6707 read counts for let-7d-3p, unpublished data) and inducing human pluripotent stem cells differentiated in the glial direction (643 read counts for miR-451a and 243 read counts for miR-1307-3p). Currently, work is underway to sequence small non-coding RNAs from the exosomes of the blood serum of the Cohort 2 patients in the present study to identify stable miRNAs that have predictive value in early and late PE onset. The data obtained will be published soon. Moreover, it was demonstrated that in a wide variety of biofluids, including serum, stability varied widely between miRNAs, with half-lives ranging from 1.5 h to more than 13 h, and was positively correlated with the sequence GC content [30]. The quantification of miR-451a and let-7d-3p was performed relative to the content of miR-1307-3p in the sample by calculating ∆Ct = Ct (miR-451a) − Ct(miR-1307-3p) and ∆Ct = Ct (let-7d-3p) − Ct(miR-1307-3p), since the highest percentage of GC in the miRNA sequence was in miR-1307-3p (73%) compared to miR-451a (36%) and let-7d-3p (50%), according to miRBase. Logistic regression models developed for predicting PE (ePE and lPE) when combining biochemical and ultrasound parameters and miR-451a, let-7d-3p, and miR-1307-3p levels are shown in Figure 6.

The best model for predicting PE in the 1st trimester of pregnancy with the lowest percentage of missed PE cases (FNR = 9.5%) and the lowest percentage of false-positive results (FPR = 15.7 %) was the combined determination of miR-451a, let-7d-3p, miR-1307-3p, UAPI, UAPI(MoM), b-hCG(MoM), and PAPPA(MoM) (Model 1 of Figure 6A). In Figure 6B, Model 1 of Figure 6A is additionally compared with Model 2, developed using only biochemical and ultrasound parameters, with the indication of formulas for calculating the probability of PE onset for both models. Figure 6B demonstrates that Model 2, using biochemical and ultrasound parameters alone, is inferior to Model 1, which uses miR-451a, let-7d-3p, and miR-1307-3p quantification in combination with biochemical and ultrasound parameters (FNR = 14.3% vs. FNR = 9.5% and FPR = 36.8% vs. FPR = 15.7% for Model 2 vs. Model 1).

### 2.3. Testing the Developed Model for Predicting PE in the First Trimester of Pregnancy on an Independent Cohort of Patients

In the next stage of our study, a third cohort of 48 women undergoing 1st trimester pregnancy screening was used to obtain a testing dataset and to evaluate the quality of the developed Model 1 from Figure 6B, obtained via a training dataset from the 2nd cohort of 40 patients. The results obtained were summarized in the form of Table 5, which also includes the results of calculating the probability of PE according to the Astraia program (https://astraia.ru/ (accessed on 1 December 2022)) and Model 2 from Figure 6B. As a rule, when using the Astraia program, the detection rate of ePE was 93.9%, that of lPE was 45.6%, and the number of false-positive results was 10.9%.

When comparing the diagnosis at the time of delivery and the established risk of PE in the 1st trimester of pregnancy for the patients from the 3rd cohort, we found that the developed formula based on miR-451a, let-7d-3p, miR-1307-3p, b-hCG(MoM), and PAPPA(MoM) serum levels and UAPI and UAPI(MoM) had better prognostic value than those based on the Astraia program, namely, the number of false-positive and false-negative cases was much lower when using Model 1 of Figure 6B in comparison with the Astraia program (FPR: 26.7 % vs. 62.1 %, FNR: 5.6 % vs. 33.3 %). It should be noted that in 4 out of 48 cases there were not enough data to calculate the probability of PE using the Astraia program. In turn, when comparing the two models (Model 1 vs. Model 2 from Figure 6B) based on the biochemical/ultrasound parameters with or without the miRNA level dataset, we confirmed the more accurate predictive value of Model 1, in particular, with FPR = 26.7 % vs. FPR = 62.0 %, and FNR = 5.6 % vs. FNR = 22.2 % (Table 5).

Therefore, the issue of the further optimization of the PE prediction model based on molecular–biological, biochemical, and instrumental methods of investigation in the first trimester of pregnancy remains relevant. One potential candidate as an additional parameter for Model 1 of Figure 6 is secretory clusterin. According to our recent study [31], the developed logistic regression models based on the level of secretory clusterin in the extravesicular fraction of the first-trimester blood serum of pregnant women have prognostic significance in assessing the probability of ePE (AUC = 0.97, Se = 1, S*p* = 0.875, cutoff = 0.3877) and lPE (AUC = 1, Se = 1, Sp = 1, cutoff = 0.5) onset. 

**Figure 7:**
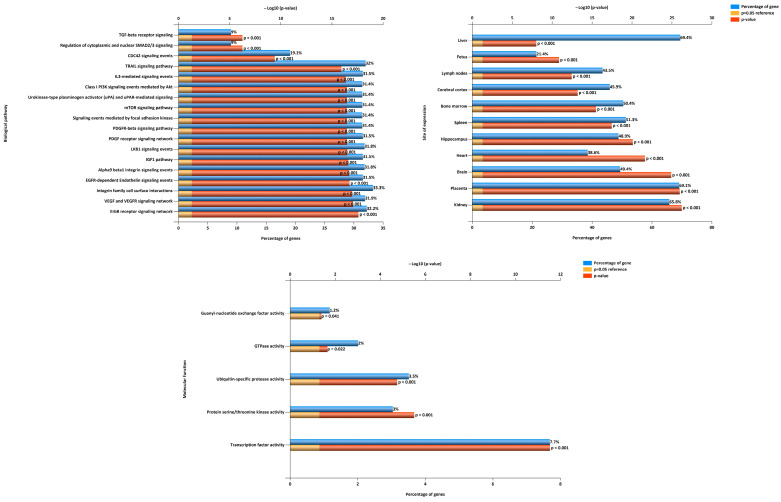
Analysis of signaling pathways influenced by miRNA predictors of ePE and lPE in the first trimester of pregnancy.

The signaling pathways, potentially regulated by the miRNA predictors of PE identified in this study (Figure 4), were analyzed using the FunRich program (Figure 7). Among them were signaling pathways with a known role in the pathogenesis of PE, namely, the ErbB, EGFR, IGF1, PDGF, TRAIL, and mTOR signaling pathways, as well as those mediated by the action of focal contact kinase and plasminogen activator urokinase type (Figure 7). Moreover, the protein products of the target genes of the identified miRNA predictors of PE were mainly regulators of transcription factor, protein serine/threonine kinase, and ubiquitin-specific protease activity.

## 3. Discussion

The search for markers for predicting PE in the first trimester of pregnancy is still ongoing due to the lack of a specific clinically significant test used in routine practice. The identification of such markers is complicated by differences in the etiopathogenesis of different forms of PE, which determine the early or late onset of clinical manifestations of PE after the 20th week of pregnancy. Back in 2008, B. Huppertz put forward a hypothesis about the pathophysiological mechanisms of PE, which are based on the impaired differentiation between villous and/or extravillous trophoblast cells [6], where the dysfunction of the two main types of trophoblast cells leads to the occurrence of PE in combination with delayed fetal development, which is typical for the early form of PE. With the overlap of the influence of maternal factors, such as genetic predisposition, immunological factors, metabolic syndrome, diabetes mellitus, and chronic arterial hypertension, the rate of onset of the clinical manifestations of PE increases [19].

Different types of miRNA species are expressed in the human placenta, in particular, those specific to trophoblasts [32,33,34]. Disbalance in the expression of sncRNAs in the placenta in early pregnancy may play a crucial role in impaired placentation. In our previous studies, we demonstrated the relationship between the profile of the embryonic secretome, namely sncRNA, and its implantation potential [35,36,37]. To track these disorders, the analysis of placenta-specific sncRNAs in maternal blood is required.

In the present study, we demonstrated the high predictive value of various combinations of let-7b-5p, miR-451a, miR-320a-3p, let-7f-5p, let-7i-5p, miR-20a-5p, miR-30e-5p, miR-22-5p, miR-320e, let-7d-3p, miR-146b-5p, miR-148a-3p, miR-519a-3p, miR-99a-5p, miR-1307-3p, miR-26a-5p, and miR-1246; according to the contents of these in the serum of women in the 1st trimester of pregnancy, it is possible to assess with high specificity and sensitivity the likelihood of developing clinical symptoms of early or late PE after 20 weeks of pregnancy (Figure 4). These miRNAs (i) were tissue-specific for placental tissue (Figure 3), (ii) had statistically significant differences in their expression levels in the placenta tissue from patients with PE at the time of delivery (Table S1, Sheet 2, and Sheet 3), (iii) made the greatest contribution to the separation of blood serum samples of pregnant women at 11–14 weeks of gestation, who later developed clinical symptoms of PE or had no signs of PE (Figure 2, Table S5), according to PLS data, and (iv) had statistically significant correlations between their content in the blood serum with either the average level of blood pressure, the uterine artery pulsation index, b-hCG, or PAPPA at 11–14 weeks of gestation (Table 3 and Table 4). Of these 17 miRNAs, according to the miRWalk database, 7 miRNAs have been proven to be involved in pre-eclampsia (miR-451a), placenta insufficiency and pre-eclampsia (miR-22-5p, miR-1246, miR-146b-5p, miR-320e, and miR-1307-3p), placenta insufficiency, vascular disease, and pre-eclampsia (miR-20a-5p) via the post-transcriptional effects on the expression of their target genes. The simultaneous detection of 3 miRNAs (let-7d-3p, miR-451a, and miR-1307-3p) resistant to the repeated freezing/thawing of blood serum samples in combination with biochemical (b-hCG and PAPP-A) and ultrasound (UAPI) parameters allowed us to develop a universal model for the prediction of ePE and lPE onset (FPR = 15. 7% and FNR = 9.5%), validated using a test cohort of patients, which demonstrated false-positive results in 26.7% of cases and false negatives in 5.6% of cases. For comparison, the use of the generally accepted Astraia program in the analysis of the test cohort of patients led to less predictive power: FPR = 62.1 % and FNR = 33.3 %.

It is well known that the let-7 family plays a key role in placental and fetal development [38,39], and its levels are controlled by the RNA-binding protein Lin28, which is a natural inhibitor of let-7 processing in both the pri- and pre-miRNA steps [40]. In turn, Lin28 is directly up-regulated via Wnt/β-catenin signaling, responsible for TE lineage specification through transcription factor Cdx2 [41]. At the same time, via the targeting of Tead4, let-7 miRNAs have an impact on the expression level of Cdx2, a clue transcription factor of the Hippo signaling pathway, which causes the development of trophectoderm through the interaction with unphosphorylated YAP1 in the nucleus of the outer cells of the blastocyst [42]. It was demonstrated that a trophectoderm-specific LIN28A/B knockdown increased the let-7 miRNA levels in blastocysts and resulted in a decrease in the expression levels of certain target genes responsible for trophoblast proliferation, invasion, migration, syncytialization, immune tolerance, angiogenesis, and vascular integrity, including the collagen genes COL1A1, COL1A2, COL3A1, COL5A1, and COL5A2; together with RAMP2, all of these genes were shown to be involved in the positive regulation of the ERK1/2 cascade, canonical Wnt- and TGF-beta signaling, and the genes of the PPAR-, PI3K-AKT-, and Hippo signaling pathways [39]. It was found that let-7 miRNA levels impacted the migration and invasion of extravillous trophoblast cells via the influence on the expression level of their common gene target MDM4 [43]. Changed levels of let-7 miRNAs were found in pregnancy-associated disorders such as pre-eclampsia [44,45]. In the present study, we found a statistically significant decrease in the expression levels of let-7f-5p, let-7c-5p, let-7b-3p, let-7i-5p, let-7a-3p, let -7d-3p, and let-7b-5p, and an increase in the expression levels of let-7e-5p and let-7e-3p in the placenta; in the blood plasma, a decrease in the content of let-7b-5p in patients with ePE at the time of delivery was found (Table S1). In the case of lPE, we found a statistically significant increase in the expression levels of let-7c-5p, let-7i-5p, let-7a-3p, let-7d-3p, let-7b-5p, and let-7d-5p, a decrease in the expression level of let-7e-5p in the placenta, and a decrease in the content of let-7e-5p and let-7a-3p in the blood plasma at the time of delivery (Table S1). That is, a different direction of changes in the expression of the genes encoding let-7c-5p, let-7i-5p, let-7a-3p, let-7d-3p, and let-7b-5p miRNAs in the placenta was revealed when comparing early and late PE in the third trimester of pregnancy, which from the viewpoint of their effects on trophoblast cell proliferation, differentiation, invasion, and migration (see above) reflects the differences in the pathogenesis of the two forms of PE. These differences are also emphasized by the fact that one of the let-7 targets is Dicer [46], which controls the biogenesis of mature miRNAs, and hence contributes to the fine tuning of the levels of their numerous target genes that form various signaling pathways in the cell. The analysis of the transcriptome of the blood serum of pregnant women at 11–13 weeks of gestation with the further development of ePE revealed decreased levels of let-7d-5p, let-7d-3p, let-7f-5p, let-7a-5p, let-7g-5p, let-7i -5p, let-7c-5p, let-7b-3p, and let-7e-5p, and increased levels of let-7b-5p and let-7a-3p, while in the case of lPE, increased levels were found for let-7e-5p, let-7g-5p, let-7f-5p, let-7a-5p, let-7c-5p, let-7i-5p, and let-7a-3p, and decreased levels were found for let-7b-5p, let-7b-3p, let-7d-5p, and let-7d-3p. Comparing ePE and lPE, a different direction of change in the concentrations of let-7f-5p, let-7a-5p, let-7g-5p, let-7i-5p, let-7c-5p, and let-7b-5p was found in the first-trimester peripheral blood serum, emphasizing the pathogenetic differences between the two PE forms even before the onset of clinical manifestations. On the contrary, the same direction of changes was found in the levels of let-7d-5p, let-7d-3p, let-7a-3p, and let-7b-3p in the first-trimester blood serum of patients with early- and late-onset PE, which indicates the presence of some common pathogenetic mechanisms for the two PE forms. For example, according to the literature, let-7d-3p affects the signaling pathways that regulate the immune response and inflammatory processes, including the signaling pathway mediated by the T-cell receptor, VEGFR, and MAPK [47]. In addition, the target gene of let-7d-3p is DNA methyltransferase 1 (DNMT1), which regulates the methylation of Notch1, PU.1, and Klf4 promoters—critical regulators of M1/M2 polarization [48].

Embryo implantation and the further development of the maternal–fetal interface depend on the differentiation of the first-trimester human placental cytotrophoblast to extravillous trophoblast via epithelial–mesenchymal transition (EMT) [49]. Among the miRNAs identified in this study as being prognostic markers of the development of early and late PE, let-7 family, miR-20a-5p, miR-451a, miR-22-5p, and miR-30e-5p are the ones that participate in EMT, according to the literature data. In particular, the role of the Wnt/β-catenin-lin28a/let-7 axis in EMT has been proven [38]. miR-20a-5p promotes EMT via the up-regulation of the expression of the mesenchymal marker vimentin and the inhibition of the expression of the epithelial marker E-cadherin via the targeting of the orphan nuclear receptor NR4A3 [50]. miR-451a is considered to be a repressor of EMT via inhibiting metalloprotease ADAM10 which regulates angiogenesis, cell migration, and proliferation [51]. miR-22 triggers EMT by inhibiting the translation of SFRP2 and PCDH15, which repress the Wnt/b-catenin pathway, essential for cell growth, invasion, and stemness [52]. miR-30e-5p inhibits cell proliferation, migration, and invasion via the targeting of snail family transcriptional repressor 1 (SNAI1), whereby the up-regulation of which is the main characteristic of EMT [53]. Additionally, it was found that the expression levels of let-7d-5p and miR-20a-5p were increased [54,55], while the expression level of let-7i-5p was decreased [56] in placental tissues or blood plasma samples collected from patients with PE. Furthermore, miR-20a-5p in combination with miR-143-3p, miR-145-5p, miR-146a-5p, miR-181a-5p, and miR-574-3p were found to be predictive molecules in assessing the development of late-onset PE via the evaluation of their content in peripheral blood leukocytes in the first trimester of pregnancy [57].

It is known that hypoxia plays a different role in placentogenesis depending on gestational age: in the first trimester of pregnancy, it promotes trophoblast invasion and angiogenesis [58], but a prolonged hypoxic condition beyond the first trimester causes deficient trophoblast syncytialization, inadequate trophoblast invasion, and impaired vascular remodeling, resulting in placental dysfunction and pregnancy-induced hypertension such as pre-eclampsia/intrauterine growth restriction [59,60]. Some miRNAs are hypoxia-responsive molecules [61,62,63], and among which are placenta-specific miRNAs, identified in the present study as being pre-eclampsia-associated markers. These include the following: mir-30e-5p, down-regulated under hypoxic conditions and causing apoptosis via the targeting of Bim [64]; decreased miR-320e, considered to be a marker of acute stroke in humans [65]; the miR-451a/MEF2D axis, affected by acidic conditions under hypoxia/ischemia, which controls cell proliferation, migration, and invasion via the Akt/GSK-3β signaling pathway [66]; the miR-451a/MIF axis, a key regulator of inflammatory processes under hypoxic conditions [67]; miR-22, induced due to myocardial ischemia-reperfusion injury and able to inhibit cardiomyocyte apoptosis via the targeting of the cAMP response element binding (CREB) protein, which regulates pro-apoptotic-related genes (Bax and p21) [68]; and miR-1307-3p, transcriptionally modulated by Hif-1α and promoting angiogenesis, cell proliferation, and invasion via the inhibition of DAB2IP and the activation of AKT/mTOR signaling [69].

Reduced utero-placental perfusion induces the placental release of antiangiogenic factors into the maternal circulation, leading to endothelial dysfunction and systemic vascular dysfunction. It was found that miR-30e-5p may serve as a circulating biomarker of microvascular dysfunction, which is associated with the targeted regulation of the genes responsible for fatty acid biosynthesis, which in turn leads to increased fatty acid β-oxidation, oxidative stress, and reduced levels of eNOS [70]. Through the knockdown of endogenous miRNAs in human endothelial cells, some of their gene targets involved in the regulation of vascular function and blood pressure were identified: FGF5 and ADRB1 for let-7b, let-7c, let-7e, and let-7g; ADRA2A and ADRA2B for miR-30e-5p; and ADM, TBX3, EDNBB, and NOX4 for miR-92a-3p [71]. It is possible that the imbalance in the levels of expression of these miRNA/mRNA pairs determines the presence of hypertensive disorders in pre-eclampsia.

It has been well established that hypoxia/ischemia in the placenta is a powerful generator of oxidative stress which stimulates the increased secretion of pro-inflammatory cytokines, such as TNFα and IL-1ß, and also results in increased trophoblastic apoptosis [72]. Oxidative stress can affect the expression levels of certain miRNAs and, conversely, miRNAs may alter the expression of the critical components of cellular antioxidants [73]. For instance, miR-93 can decrease Nrf2, which activates the transcription of genes coding antioxidant enzymes [74]. In turn, Nrf2 binds to the miR-1246 gene promoter, ensuring its expression [75]. A significant increase in Nrf2 and miR-1246 gene expression during human syncytiotrophoblast differentiation and a marked decrease in their expression levels in the placentas of women with severe pre-eclampsia were revealed [75]. It is important to note that miR-1246 target genes are inhibitors of WNT/b-catenin signaling (GSK3b and AXIN2), which are crucial for placental development. This finding underlines the importance of using miR-1246 in our logistic regression model for predicting the development of early and late pre-eclampsia during the first screening of pregnant women.

There is a close relationship between ER stress and oxidative stress in the pathogenesis of pre-eclampsia [72]: two types of intracellular stress are caused by ischaemia-reperfusion and hypoxia, and an unfolded protein response (UPR) in the case of the accumulation of misfolded proteins can activate some of the same intracellular inflammatory signaling pathways as oxidative stress (NF-κB and p38MAPK pathways). In the present study, we identified miR-1307-3p as being an early predictive marker of pre-eclampsia, having the potential to target methyltransferase protein 8 (METTL8), which in turn inhibits the expression of KDM3A/3B and reduces methylation on the ninth lysine of histone H3, therefore providing the binding ability of P300 and RNA pol II to the promoter region of endoplasmic reticulum UPR effector protein CALR [76]. We found a decrease in the miR-1307-3p level in the first-trimester blood serum level of women with early or late PE (Table S4), which indicates the impaired functioning of the endoplasmic reticulum UPR-signaling even before the onset of clinical manifestations of PE. At the time of delivery, the placental tissue from women with ePE had a reduced level of miR-1307-3p, and in the case of lPE, there was an increased level of miR-1307-3p (Table S1), probably indicating the elimination of compensatory mechanisms of endoplasmic reticulum stress and later manifestations of PE symptoms in comparison with ePE. In our recent study, we analyzed the blood serum of pregnant women for the level of secretory clusterin, which is an intra- and extracellular chaperone and is involved in the processes induced by ER stress [31]. A high degree of prediction of the development of early and late PE via the level of secretory clusterin was revealed long before the onset of clinical manifestations of these pregnancy complications.

Thus, the placenta-specific miRNAs identified in this study as being markers for predicting the development of early and late PE in the preclinical stage of the disease are involved in the main processes of its pathogenesis, namely, impaired differentiation; proliferative and invasive abilities of cytotrophoblast cells, leading to the abnormal remodeling of the spiral uterine arteries and an increase in their resistance, causing hypoxic-ischemic damage of the placental tissue and the occurrence of oxidative/endoplasmic reticulum stress; inflammatory reactions; systemic endotheliosis; and multiple organ dysfunction/insufficiency. Obviously, the better we understand the pathogenesis of pre-eclampsia and the earlier it is diagnosed, the wider the range of drugs there will be with the potential to treat pre-eclampsia and the higher the effectiveness of these drugs will be. Low-dose aspirin is currently the only preventive medication recommended for patients with an elevated risk of pre-eclampsia. However, hydroxychloroquine is proposed as a promising drug in preventing and treating pre-eclampsia as it promotes anti-inflammatory, anti-oxidant, and anti-thrombotic effects [77].

## 4. Materials and Methods

### 4.1. Patients

A total of 115 patients aged 25–40 were selected from those admitted to the National Medical Research Center for Obstetrics, Gynecology, and Perinatology, named after the Academician V.I. Kulakov of Ministry of Healthcare of the Russian Federation for Management of Pregnancy and Delivery, and signed informed consent to participate in the study was obtained; the study was approved by the Ethics Committee of the Center. Clinical and biochemical blood tests, ultrasound examination of the pelvic and fetal organs, feto-placental blood flow Dopplerometry, cardiotocography, blood pressure measurement, the determination of protein levels in urine, and concentrations of PLGF, sFlt-1, PAPP-A, and β-HCG in blood serum using diagnostic test systems were carried out for each patient. The criteria for non-inclusion in the study were the onset of pregnancy via assisted reproductive technologies, multiple pregnancy, and fetal aneuploidy.

### 4.2. RNA Isolation from Blood Plasma or Serum

A total of 200 µL of blood plasma or serum, purified from cells and cell debris via stepwise centrifugations at 300× g for 20 min and at 16,000× g for 10 min, were used for RNA isolation using an miRNeasy Serum/Plasma kit (Qiagen, Hilden, Germany) following the preliminary addition of 5.6x108 copies of synthetic RNA cel-miR-39 (Qiagen, Hilden, Germany) after plasma/serum incubation with QIAzol Lysis Reagent (Qiagen, Hilden, Germany) to control the efficiency of RNA extraction and cDNA synthesis in accordance with the manufacturer’s recommendations.

### 4.3. RNA Isolation from Placenta Tissue

Placental tissue samples were taken for research no later than 10 min after delivery. A 5 mm thick tissue slice was taken via passing an instrument through the entire placenta from the fetal surface down through the maternal surface at a location roughly halfway between the umbilical cord attachment site and the placental margin in an area free from any obvious abnormalities, as recommended by Burton GJ et al. [78]. The sampled placental tissue free of fetal membranes was washed in 0.9% NaCl and immediately frozen in liquid nitrogen for subsequent storage at −80 °C. Total RNA was extracted from 20 to 40 mg of placental tissue using the miRNeasy Micro Kit (Qiagen, Hilden, Germany) followed by the RNeasy MinElute Cleanup Kit (Qiagen, Hilden, Germany). The RNA concentration was measured using a Qubit fluorimeter 3.0 (Life Technologies, Petaling Jaya, Malaysia). The sample quality of the total RNA was examined using the Agilent Bioanalyzer 2100 (Agilent, Waldbronn, Germany) and the RNA 6000 Nano Kit (Agilent Technologies, Santa Clara, CA, USA). Total RNA samples with a 28S/18S ribosomal RNA ratio equal to 1.5–1.8 were used for further studies.

### 4.4. miRNA Deep Sequencing

cDNA libraries were synthesized using 6 µL of total RNA column eluate (miRNeasy Serum/Plasma Kit) extracted from 200 µL of blood serum or plasma and 500 ng of total RNA from placental tissue using the NEBNext^®^ Multiplex Small RNA Library Prep Set for Illumina^®^ (Set11 and Set2, New England Biolab^®^, Frankfurt am Main, Germany, cat. nos. E7300S and E7580S), amplified for 19 and 14 PCR cycles, respectively, and sequenced using the NextSeq 500 platform (Illumina, San Diego, CA, USA, cat. no. SY-415-1001). The adapters were removed using Cutadapt. All trimmed reads shorter than 16 bp and longer than 55 bp were filtered, and only reads with a mean quality higher than 15 were retained. The remaining reads were mapped to the GRCh38.p15 human genome and miRBase v21 using the bowtie aligner [79]. Aligned reads were counted using the featureCount tool from the Subread package [80] and the fracOverlap 0.9 option; so, the whole read was forced to have a 90% intersection with sncRNA features. Differential expression analysis of the sncRNA count data was performed using the DESeq2 package [81].

### 4.5. Reverse Transcription and Quantitative Real-Time PCR

Five microliters from fourteen µL of total RNA column eluate (miRNeasy Serum/Plasma Kit, Qiagen, Hilden, Germany) extracted from two hundred µL of blood serum was converted into cDNA in accordance with the miScript^®^ II RT Kit protocol (Qiagen, Hilden, Germany); then, the sample volume was adjusted to 200 µL using deionized water. The synthesized cDNA (2 µL) was used as a template for real-time PCR using a forward primer specific for the studied miRNA (Table 6) and the miScript SYBR Green PCR Kit (Qiagen, Hilden, Germany). The following PCR conditions were used: (1) 15 min at 95 °C and (2) 50 cycles at 94 °C for 15 s, an optimized annealing temperature (52–62 °C) for 30 s, and 70 °C at 30 s in a StepOnePlusTM thermocycler (Applied Biosystems, Waltham, MA, USA). The relative expression of miRNA in the blood serum samples was determined via the ∆Ct method, using cel-39 as the reference RNA.

### 4.6. Statistical Analysis of the Obtained Data

For statistical processing, scripts written in R language [80] and RStudio [82] were used. The correspondence of the analyzed parameters to the normal distribution law was assessed via the Shapiro–Wilk test. When the distribution of data was different from normal, the Mann–Whitney test for paired comparison was used. Since both quantitative and qualitative characteristics were analyzed, a rank-order correlation analysis was performed using Spearman’s non-parametric correlation test. The 95% confidence interval for the correlation coefficient was determined using Fisher transformation. The value of the threshold significance level (*p*) was taken as being equal to 0.05.

## 5. Conclusions

A universal model for the prediction of ePE and lPE onset via the simultaneous detection of cell-free placenta-specific miRNAs (let-7d-3p, miR-451a, and miR-1307-3p) resistant to the repeated freezing/thawing of blood serum samples in combination with biochemical (b-hCG and PAPP-A) and ultrasound (UAPI) parameters in the first trimester of pregnancy was developed using a training cohort of 40 patients (FPR = 15.7% and FNR = 9.5%), and was validated using a test cohort of 48 patients with false-positive results in 26.7% of cases and false negatives in 5.6% of cases. For comparison, the use of the generally accepted Astraia program in the analysis of the test cohort of patients led to less predictive results: FPR = 62.1% and FNR = 33.3%. The participation of these miRNAs in trophoblast proliferation, invasion, migration, syncytialization, the endoplasmic reticulum unfolded protein response, immune tolerance, angiogenesis, and vascular integrity according to the literature data underlines the significance of their use as prognostic molecules for revealing PE in the first-trimester screening of pregnant women.

## Figures and Tables

**Table 1:** Clinical characteristics of the first cohort of patients.

	Normal Pregnancy	Complicated Pregnancy		
Delivery	Planned Caesarean Section	Emergency Caesarean Section Because of the Risk of Early Pregnancy Failure	Caesarean Section Because of Early Pre-Eclampsia	Planned Caesarean Section Because of Late Pre-Eclampsia
Group of pregnant women (number of patients)	N > 34 (*n* = 6)	N < 34 (*n* = 7)	ePE (*n* = 7)	lPE (*n* = 7)
Pre-eclampsia manifestation time (weeks)	No	No	24.5 (22.0; 28.0) *	36.1 (36.0; 37.0) *
Delivery time (weeks)	38.0 (37.0; 39.0) *	29.0 (25.0; 32.0) *	28.2 (25.0; 30.0) *	36.9 (36.0; 38.0) *
Severe pre-eclampsia (number of patients)	0	0	7	1
Mild pre-eclampsia (number of patients)	0	0	0	6
Edema of legs and feet (number of patients)	0	0	1	5
Urine protein level (0.0–0.2 g/L)	Normal	Normal	2.3 (0.2; 4.6) *	1.4 (0.1; 4.1) *
Blood pressure:				
systolic	112 (107; 119) *	116 (112; 120) *	155 (125; 180) *	144 (120; 175) *
diastolic	68 (65; 71) *	77 (74; 81) *	100 (80; 120) *	93 (70; 100) *
Alanine-aminotransferase, ALT (up to 31.0 U/L)	No data	No data	74 (11; 215) *	23 (12; 32) *
Aspartate aminotransferase, AST (up to 31.0 U/L)	No data	No data	55 (11; 194) *	29 (16; 48) *
Alkaline phosphatase (up to 239.0 U/L)	No data	No data	110 (54; 179) *	165 (79; 252) *
Platelets of peripheral blood (150–390 thou/mm^3^)	228 (166; 290) *	238 (183; 293) *	145 (68; 243) *	238 (181; 308) *
PLGF (54–862 pg/mL) for 37–40 GW [29]	No data	No data	30 (14; 47) *	101 (54; 216) *
sFLT-1 (1533–9184 pg/mL) for 37–40 GW [29]	No data	No data	11957 (5615; 23,226) *	14657 (7489; 24,990) *
sFLT-1/PLGF (<110) [29]	No data	No data	444 (126; 847) *	193 (42; 348) *

Note: *—all data are given as means (minimum; maximum).

**Table 2:** Clinical characteristics of the second cohort of patients, screened in the first trimester of pregnancy.

	Physiological Full-Term Pregnancy, N (*n* = 10)	Pregnancy with High sFLT-1/PLGF Ratio without Signs of PE, Nhr (*n* = 9)	lPE (*n* = 10)	ePE (*n* = 11)
	First pregnancy trimester screening			
Gestational age	12.5 (12.0; 13.4)	12.1 (11.2; 13.1)	12.2 (11.6; 12.5)	12.0 (11.2; 12.4)
Crown-rump length, CRL (43.0–84.0 mm)	62.5 (54.0; 74.7)	59.1 (50.0; 69.0)	59.6 (55.1; 64.0)	57.4 (50.0; 62.0)
Nuchal translucency thickness, NT (1.6–1.7 mm)	1.4 (1.1; 2.2)	1.5 (1.0; 2.0)	1.6 (1.3; 2.0)	1.7 (1.1; 2.9)
Uterine artery pulsatility index, UA (PI), 0.9–2.6 (5th and 95th percentiles)	1.6 (0.4; 2.2)	1.8 (1.2; 2.5)	1.7 (0.7; 2.4)	2.1 (1.3; 3.5)
UA (PI) MoM	0.9 (0.3; 1.3)	1.1 (0.8; 1.4)	1.0 (0.4; 1.5)	1.1 (0.3; 2.1)
b-hCG (50.0–55.0 IU/mL)	68.7 (52.3; 89.8)	47.1 (23.1; 114.6)	36.4 (27.8; 53.6)	43.4 (15.6; 94.3)
b-hCG (0.5–2.0 MoM)	1.5 (1.1; 2.3)	1.1 (0.4; 2.5)	0.8 (0.5; 1.6)	0.9 (0.3; 1.7)
PAPP-A (0.7–6.0 IU/L)	3.1 (1.6; 6.9)	2.4 (1.1; 4.2)	2.7 (0.6; 5.0)	2.4 (0.8; 6.2)
PAPP-A (0.5–2.0 MoM)	1.2 (0.5; 3.2)	1.2 (0.4; 2.4)	0.9 (0.4; 2.7)	1.1 (0.5; 2.9)
	Delivery			
Gestational age	38.6 (36.0; 40.6)	37.7 (31.0; 40.2)	37.3 (35.4; 38.5)	31.9 (28.2; 35.6)
Alanine-aminotransferase, ALT (up to 31.0 U/L)	31.8 (8.8; 95.0)	24.1 (11.8; 46.1)	34.2 (12.4; 165.1)	78.2 (11.8; 352.2)
Aspartate aminotransferase, AST (up to 31.0 U/L)	19.7 (11.1; 25.5)	24.3 (13.0; 40.5)	48.9 (10.9; 262.3)	68.7 (13.6; 282.4)
Alkaline phosphatase (up to 239.0 U/L)	182.3 (130.8; 292.6)	130.2 (94.2; 183.0)	208.8 (154.3; 319.6)	119.8 (87.1;169.2)
Lactate dehydrogenase, LDH (130.0–220.0 U/L)	345.6 (271.0; 408.2)	362.2 (296.8; 422.2)	435.8 (36.4; 743.1)	598.7 (351.4;1680.0)
BP systolic (20 to 40 years; 120-127 mm Hg)	118 (90; 140)	135 (105; 170)	140 (127; 160)	152 (140; 170)
BP diastolic (75–80 mm Hg)	77 (60; 90)	86 (70; 110)	91 (80; 105)	98 (90; 110)
Protein level in urine (0.0–0.2, g/L)	0.1 (0.1; 0.1)	0.1 (0.0; 0.1)	0.4 (0.2; 0.9)	2.1 (0.2; 3.4)
Peripheral blood leukocytes (4.0–9.0 thou/mm^3^)	9.5 (5.2; 15.6)	8.8 (7.8; 10.5)	10.8 (7.7; 24.4)	11.5 (3.3; 23.2)
Platelets of peripheral blood (150–390 thou/mm^3^)	261.8 (201.0; 390.0)	209.9 (146.0; 287.0)	210.1 (93.0; 300.0)	203.5 (82.0; 359.0)
PLGF (54–862 pg/mL) for 37–40 GW [29]	115.3 (94.4; 143.8)	74.9 (43.2; 113.4)	83.6 (34.2; 152.0)	56.8 (22.2; 109.7)
sFLT-1 (1533–9184 pg/mL) for 37–40 GW [29]	6271.0 (5168.0; 7763.0)	11895.6 (5190.0; 19418.0)	9651.7 (4027.0; 14131.0)	10722.8 (5216.0; 19738.0)
sFLT-1/PLGF (<110) [29]	54.4 (53.9; 54.8)	173.4 (107.3; 430.1)	129.4 (66.8; 233.6)	285.4 (48.8; 636.2)
Edema of legs and feet (number of patients)	3	1	4	5
Full-term fetus weight, 3200–3500 g	3396.5 (2880.0; 3952.0)	2764.4 (780.0; 3550.0)	2744.9 (2132.0; 3518.0)	1424.2 (900.0; 2582.0)
Placenta weight at full-term pregnancy, 390–415 g	463.1 (303.0; 650.0)	324.4 (106.0; 449.0)	370.3 (257.0; 465.0)	230.2 (119.0; 371.0)
Mean uterine artery PI (39th week, 5th and 95th percentiles: 0.47–0.91)	0.6 (0.5; 0.7)	0.8 (0.5; 1.7)	0.9 (0.6; 1.1)	1.2 (1.0; 1.5)
Umbilical artery PI (39th week, 5th and 95th percentiles: 0.76–1.03)	0.8 (0.6; 1.4)	1.1 (0.7; 2.3)	0.9 (0.7; 1.0)	1.4 (0.8; 1.8)
Middle cerebral artery PI (39th week, 5th and 95th percentiles: 0.93–1.73)	1.4 (1.2; 1.7)	1.4 (1.2; 1.6)	1.3 (0.6; 1.7)	1.6 (1.1; 2.4)
Cerebro/placental ratio, > 1	1.8 (1.1; 2.5)	1.4 (0.6; 2.0)	1.5 (1.1; 2.3)	1.3 (0.8; 1.9)

Note: all data except for “edema of legs and feet” are given as means (minimum; maximum).

**Table 3:** Correlation analysis of blood serum miRNA levels with mean arterial blood pressure in pregnant women in the first trimester via non-parametric Spearman method, and involvement of miRNA target genes in the pathogenesis of various diseases according to miRWalk database.

	Mean Arterial Blood Pressure		miRWalk Database (Disease ID)		
	r	*p*	DOID:10825 #Essential Hypertension	DOID:1591 #Renovascular Hypertension	DOID:10591 #Pre-Eclampsia
hsa-miR-615-3p	0.51	0.0009	x	x	x
hsa-miR-16-2-3p	0.49	0.0014		x	
hsa-miR-107	0.47	0.0022	x	x	x
hsa-miR-320a	0.45	0.0036			
hsa-miR-182-5p	0.44	0.0049	x		x
hsa-miR-320b	0.44	0.0048	x	x	x
hsa-miR-92b-3p	0.44	0.005	x	x	x
hsa-miR-101-3p	0.42	0.0069	x	x	
hsa-miR-10b-5p	0.42	0.0074	x	x	x
hsa-miR-1304-5p	0.42	0.007	x	x	x
hsa-miR-185-5p	0.41	0.0086	x	x	x
hsa-miR-3613-5p	0.4	0.0115			
hsa-miR-25-3p	0.39	0.0138		x	x
hsa-miR-451a	0.39	0.012			x
hsa-miR-144-3p	0.37	0.0191			
hsa-miR-125a-5p	0.36	0.0218	x	x	x
hsa-miR-183-5p	0.36	0.0217	x	x	x
hsa-miR-139-3p	0.35	0.0258	x		x
hsa-miR-320c	0.35	0.029	x	x	x
hsa-miR-363-3p	0.35	0.0291		x	x
hsa-miR-652-3p	0.34	0.0317	x	x	x
hsa-miR-92a-3p	0.34	0.0297	x	x	x
hsa-miR-378c	0.33	0.0381	x	x	x
hsa-let-7c-5p	0.32	0.046	x	x	x
hsa-miR-15a-5p	0.32	0.0436	x		x
hsa-miR-4732-5p	0.32	0.0476	x	x	x
hsa-miR-148a-5p	0.31	0.0511		x	x
hsa-miR-381-3p	−0.31	0.0518	x	x	x
hsa-miR-99b-3p	−0.32	0.0444	x	x	x
hsa-miR-340-5p	−0.33	0.036			
hsa-miR-134-5p	−0.34	0.0345	x	x	x
hsa-miR-17-5p	−0.34	0.0345	x	x	x
hsa-miR-493-3p	−0.34	0.0329	x	x	x
hsa-miR-330-3p	−0.35	0.0274		x	x
hsa-miR-323a-3p	−0.36	0.0213			x
hsa-miR-503-5p	−0.37	0.0198		x	x
hsa-miR-323b-3p	−0.38	0.0159	x	x	x
hsa-miR-374a-5p	−0.39	0.0135			
hsa-miR-382-5p	−0.39	0.0119	x	x	x
hsa-miR-335-5p	−0.42	0.007			
hsa-miR-199b-5p	−0.47	0.0023	x	x	x

**Table 4:** Data from non-parametric Spearman correlation analysis of miRNA levels in blood serum and clinical/laboratory data on first-trimester pregnancy screening, and participation of miRNA target genes in the pathogenesis of various diseases according to miRWalk database.

miRNA	Uterine Artery Pulsatility Index (UAPI)		miRWalk Database (Disease ID)		
	r	*p*	DOID:3891 #Placental Insufficiency	DOID:178 #Vascular Disease	DOID:10591 #Pre-Eclampsia
hsa-miR-22-5p	−0.48	0.0017	x		x
hsa-miR-20a-5p	0.41	0.0082	x	x	x
hsa-miR-942-5p	−0.4	0.0097	x		x
hsa-miR-125b-5p	0.32	0.0443	x		x
hsa-miR-1-3p	0.31	0.0493			
hsa-miR-150-3p	−0.31	0.053	x	x	x
	UAPI_MoM				
	r	*p*			
hsa-miR-425-3p	0.42	0.0077			x
hsa-miR-6087	0.4	0.0096			
hsa-miR-20a-5p	0.4	0.01	x	x	x
hsa-miR-204-5p	−0.34	0.0295	x		x
hsa-miR-1-3p	0.33	0.0356			
hsa-miR-126-5p	−0.33	0.0386			
hsa-miR-885-5p	0.32	0.0448	x		x
hsa-miR-520a-3p	−0.32	0.0458	x		x
hsa-miR-942-5p	−0.31	0.0503	x		x
hsa-miR-1246	0.31	0.0526	x		x
	b-HCG_MU				
	r	*p*			
hsa-miR-326	0.39	0.0132	x	x	x
hsa-miR-106b-5p	−0.38	0.0153	x	x	x
hsa-miR-760	0.38	0.016	x		x
hsa-miR-193b-5p	0.35	0.0278	x	x	x
hsa-miR-3605-3p	0.34	0.0314	x		x
	b-HCG_MoM				
	r	*p*			
hsa-miR-326	0.58	0.0001	x	x	x
hsa-miR-760	0.45	0.0039	x		x
hsa-miR-193b-5p	0.42	0.0068	x	x	x
hsa-miR-522-3p	0.35	0.0252	x		x
hsa-miR-106b-5p	−0.35	0.0289	x	x	x
hsa-miR-378a-3p	0.33	0.0382	x		x
hsa-miR-130a-3p	0.32	0.0445	x	x	x
	PAPP-A_MU				
	r	*p*			
hsa-miR-1-3p	−0.4	0.0108			
hsa-miR-146b-5p	−0.38	0.0156	x		x
hsa-miR-664a-5p	0.36	0.0209	x		x
hsa-miR-615-3p	−0.36	0.0228	x	x	x
hsa-miR-320e	0.36	0.0232	x		x
hsa-miR-942-5p	0.35	0.0262	x		x
hsa-miR-652-3p	−0.32	0.0472	x		x
hsa-miR-335-3p	0.31	0.0497			x
	PAPP-A_MoM				
	r	*p*			
hsa-miR-517a-3p	0.39	0.0121	x		x
hsa-miR-517b-3p	0.39	0.0121	x		x
hsa-miR-1307-3p	0.38	0.0164	x		x
hsa-miR-223-3p	0.38	0.0168	x		x
hsa-miR-425-3p	−0.35	0.026			x
hsa-miR-942-5p	0.35	0.026	x		x
hsa-miR-140-3p	0.35	0.028			x
hsa-miR-320e	0.33	0.0351	x		x
hsa-miR-30b-5p	-0.33	0.0365		x	x
hsa-miR-127-3p	-0.33	0.0387	x		x
hsa-miR-493-5p	-0.32	0.0475	x		x
hsa-miR-126-5p	0.31	0.0487			
hsa-miR-1323	0.31	0.0532		x	x

**Table 5:** Calculation of the probability of PE onset based on Astraia program algorithm and on the formulas of Models 1 and 2 of Figure 6B while using a test cohort of pregnant women undergoing first-trimester screening.

Sample ID	Risk of PE According to the Astraia Program	Model 2 of Figure 6B: UAPI, UAPI(MoM), b-hCG(MoM), and PAPPA(MoM); Threshold = 0.508		Model 1 of Figure 6B: miR-451a, let-7d-3p, miR-1307-3p, UAPI, UAPI(MoM), b-hCG(MoM), and PAPPA(MoM); Threshold = 0.52		Diagnosis at Delivery
60	PE	0.513	PE	0.720	PE	No symptoms of PE up to delivery. sFlt/PLGF = 41.01 at a rate of up to 29.8.
61	N	0.988	PE	0.984	PE	No symptoms of PE up to delivery. bHCG = 227.4 at a rate of up to 121 for 11–14 GW. sFlt/PLGF = 5.62 at a rate of up to 8.8.
62	PE	0.833	PE	0.075	N	Physiological term pregnancy.
63	PE	0.334	N	0.010	N	Physiological term pregnancy.
64	PE	0.434	N	0.021	N	Physiological term pregnancy.
65	PE	0.864	PE	0.956	PE	No symptoms of PE up to delivery. GDM, gestational oedema, hypothyroidism, hereditary thrombophilia, 1st-degree violation of the utero-placental blood flow at 17 GW, isthmic-cervical insufficiency. sFlt/PLGF = 46.74 at a rate of up to 52.4.
66	N	0.660	PE	0.013	N	Physiological term pregnancy.
67	ND *	0.513	PE	0.743	PE	No symptoms of PE up to delivery. Multigenic thrombophilia, recurrent abortion, isthmic-cervical insufficiency, violation of the fetal–placental blood flow (Type 1). According to Astraia, the risk of IUGR was 1:156. sFlt/PLGF = 3.39 at a rate of up to 8.8.
68	N	0.230	N	0.008	N	No symptoms of PE up to delivery. GAG.
69	PE	0.463	N	0.067	N	No symptoms of PE up to delivery. GAG.
70	N	0.525	PE	0.216	N	No symptoms of PE up to delivery. GAG.
71	N	0.237	N	0.047	N	No symptoms of PE up to delivery. GAG.
72	PE	0.551	PE	0.188	N	No symptoms of PE up to delivery. CAG.
73	PE	0.312	N	0.087	N	No symptoms of PE up to delivery. CAG.
74	PE	0.634	PE	0.719	PE	No symptoms of PE up to delivery. CAG, isthmic-cervical insufficiency, GDM, 1st-degree violation of the utero-placental blood flow, recurrent abortion, sFlt/PLGF = 87.49 at a rate of up to 29.8.
75	PE	0.291	N	0.976	PE	No symptoms of PE up to delivery. CAG, history of transient ischemic attacks, hypothyroidism, hereditary thrombophilia, GDM, augmenting of gestational oedema from 34 GW. sFlt/PLGF = 63.11 at a rate of up to 52.4.
77	N	0.491	N	0.222	N	No symptoms of PE up to delivery. IUGR.
78	N	0.416	N	0.139	N	No symptoms of PE up to delivery. CAG.
79	PE	0.901	PE	0.730	PE	No symptoms of PE up to delivery. IUGR, oligohydramnios, 2nd-degree violation of the utero-placental blood flow, sFlt/PLGF = 143.03 at a rate of up to 52.4.
80	N	0.446	N	0.124	N	No symptoms of PE up to delivery. GAG.
82	N	0.992	PE	1.000	PE	No symptoms of PE up to delivery. Intrauterine fetal death at 27 weeks (fetal weight 300 g at a rate of 758 ± 227, small placenta for gestational age). According to Astraia, the risk of IUGR was 1:110. Shortening of the tubular bones of the fetus from 17 GW. Third-degree violation of the utero-placental blood flow and third-degree violation of the feto-placental blood flow at twenty-four GW.
83	PE	0.541	PE	0.001	N	No symptoms of PE up to delivery. GAG.
84	PE	0.842	PE	0.090	N	No symptoms of PE up to delivery. GAG.
85	N	0.315	N	0.261	N	No symptoms of PE up to delivery. GAG.
96	PE	0.781	PE	0.018	N	Physiological term pregnancy.
99	N	0.599	PE	0.003	N	Physiological term pregnancy.
100	PE	0.541	PE	0.143	N	No symptoms of PE up to delivery. IUGR, CAG.
107	PE (1:4)	0.744	PE	0.222	N	No symptoms of PE up to delivery. IUGR, CAG, antiphospholipid syndrome, autoimmune thyroiditis, hypothyroidism, 3rd-degree violation of the utero-placental blood flow and 1st-degree violation of the feto-placental blood flow at 30 GW, decreased cerebro/placental ratio. sFlt/PLGF = 231.2 at a rate of up to 8.8.
108	PE	0.741	PE	0.047	N	No symptoms of PE up to delivery. IUGR, CAG, antiphospholipid syndrome, autoimmune thyroiditis, hypothyroidism, 3rd-degree violation of the utero-placental blood flow and 2nd-degree violation of the feto-placental blood flow at 26 GW, decreased cerebro/placental ratio. Autosomal dominant polycystic kidney disease, chronic kidney disease stage 1. According to Astraia, the risk of IUGR was 1:4. sFlt/PLGF = 524.49 at a rate of up to 8.8.
87	PE	0.630	PE	0.446	N	No symptoms of PE up to delivery. Chronic pyelonephritis. Gestational oedema. GDM. sFlt/PLGF = 80.48 at a rate of up to 52.4.
81	N	0.924	PE	0.902	PE	Moderate lPE
86	PE	0.752	PE	0.998	PE	Moderate lPE
88	PE	0.971	PE	0.976	PE	Moderate ePE, CAG
89	PE	0.718	PE	0.994	PE	Severe ePE, IUGR
90	PE	0.564	PE	0.970	PE	Severe lPE
91	ND *	0.972	PE	1.000	PE	Moderate lPE
92	PE	0.946	PE	0.925	PE	Severe lPE
93	PE	0.462	N	0.564	PE	Moderate lPE
94	PE	0.478	N	0.570	PE	Moderate lPE
95	PE	0.551	PE	0.990	PE	Moderate ePE, CAG
97	ND *	0.477	N	0.235	N	Moderate lPE, GAG
98	N	0.878	PE	0.998	PE	Moderate lPE
101	N	0.899	PE	0.911	PE	Moderate lPE
103	N	0.587	PE	0.997	PE	Moderate lPE
104	N	0.775	PE	0.860	PE	Moderate lPE
105	PE	0.523	PE	0.640	PE	Moderate lPE, CAG
106	PE	0.284	N	0.966	PE	Severe ePE, CAG
109	ND *	0.583	PE	0.920	PE	Severe ePE

* ND—non-determined value.

**Table 6:** Sequence characteristics of miRNAs analyzed via real-time PCR.

miRNA	miRBase ID	Nucleotide Sequence of Forward PCR Primer (5′-3′)	Annealing Temperature (°C)
miR-451a	MIMAT0001631	aaaccgttaccattactgagtt	55
let-7b-5p	MIMAT0000063	tgaggtagtaggttgtgtggtt	60
miR-320a-3p	MIMAT0000510	aaaagctgggttgagagggcga	60
let-7i-5p	MIMAT0000415	tgaggtagtagtttgtgctgtt	52
let-7f-5p	MIMAT0000067	tgaggtagtagattgtatagtt	51.3
miR-20a-5p	MIMAT0000075	taaagtgcttatagtgcaggtag	51.3
miR-30e-5p	MIMAT0000692	tgtaaacatccttgactggaag	52.7
miR-22-5p	MIMAT0004495	agttcttcagtggcaagcttta	52.7
miR-320e	MIMAT0015072	aaagctgggttgagaagg	48.9
miR-146a-5p	MIMAT0000449	tgagaactgaattccatgggtt	54
miR-192-5p	MIMAT0000222	ctgacctatgaattgacagcc	59
miR-10b-5p	MIMAT0000254	taccctgtagaaccgaatttgtg	58.7
miR-128-3p	MIMAT0000424	tcacagtgaaccggtctcttt	59
miR-16-5p	MIMAT0000069	tagcagcacgtaaatattggcg	62
miR-484	MIMAT0002174	tcaggctcagtcccctcccgat	62
miR-17-5p	MIMAT0000070	caaagtgcttacagtgcaggtag	55
miR-25-3p	MIMAT0000081	cattgcacttgtctcggtctga	56
miR-92a-3p	MIMAT0000092	tattgcacttgtcccggcctgt	60
miR-93-5p	MIMAT0000093	caaagtgctgttcgtgcaggtag	55
let-7d-3p	MIMAT0004484	ctatacgacctgctgcctttct	51.3
miR-99a-5p	MIMAT0000097	aacccgtagatccgatcttgtg	55
miR-519a-3p	MIMAT0002869	aaagtgcatccttttagagtgt	52
miR-1307-3p	MIMAT0005951	actcggcgtggcgtcggtcgtg	46.2
miR-26a-5p	MIMAT0000082	ttcaagtaatccaggataggct	51.2
miR-1246	MIMAT0005898	aatggatttttggagcagg	57.6

## Data Availability

Not applicable.

## Supplementary Materials

The following supporting information can be downloaded at https://www.mdpi.com/article/10.3390/ijms24098006/s1.
